# Management of the Specific Remedy

**Published:** 1882-10

**Authors:** P. P. Wells

**Affiliations:** Brooklyn, New York


### HOMŒOPATHIC PHYSICIAN.

A MONTHLY JOURNAL OF MEDICAL SCIENCE.

"If our school ever gives up the strict inductive method of Hahnemann, we are lost, and deserve only to be mentioned as a caricature, in the history of medicine."—CONSTANTINE HERING.

Vol. II. OCTOBER, 1882. No. 10.

### MANAGEMENT OF THE SPECIFIC REMEDY.

P. P. WELLS M. D., BROOKLYN, N. Y.

The first duty of the practicing homœopathician is to find the specific remedy for the case under his hand. The second is but little, if at all, less important, and that is its right administration when found. The great burden of the utterances of those who have written best on practical homœopathic duty, has been how to discharge this first duty. How shall the specific be found? On this there has been much and well said, so much that perhaps more is not needed. But, the specific being found, "What shall we do with it?" has not been so fully discussed and answered. Instead of this, the latest and most important practical question related in any degree to this, is "*What shall we do with Homœopathy?*" This is said to be occupying the attention of our friends of "the old school."* It is certainly well that it should do so. Perhaps no question is more worthy of their serious attention. If it be true that "old school" physic is now seriously considering this question, it must be not a little perplexing to it when it finds, in so many instances, where its professed advocates are assembled in any considerable numbers, in society, convention, or institute, these are so often found most earnest and busy in putting it out of sight.† This is just what too many of these bodies seem most anxious to do, and to be most busy in doing. The name—it is of no value.‡ Its

---

**Medical Times*, Vol. x, No. 5, p. 153.

† See last resolution of the American Institute.

‡ *Medical Times, loc. cit.*

philosophy, it seems but a trouble, and to be wholly distasteful to them, and therefore is but little, if at all, understood. Indeed, judging by the utterances of these bodies, it may at times be a question whether they have a thought that there is any such thing as a philosophy of Homœopathy. If it be otherwise, then we have resolutions approving of its neglect, or whatever of violations of its principles or rules of practice any of the members may find a convenience in enacting. The law—why don't they "*stand like a rock upon it.*"* It is submitted better practical results will come from *obeying* law, than from "*standing on*" it. But this *standing on* is about all the relation these people have to this law. As a thing to stand on—well, it seems to be about all the knowledge they have of it. The law—it is troublesome. "To obey it at all times it is most difficult." Well, what then? And yet we hear, and often from those who turn their backs on the law most frequently: "Shall we let our patients die for the sake of adherence to a dogma?" No! by no means. Rather give them the best possible chance for their lives by a strict obedience to the law God has given for their healing. If their best chance for life is not in this, then the law on which these men are so earnest in standing is no law, and God has had nothing to do in its enactment.

This "not leaving a patient to die for the sake of a dogma" seems to address itself to the complacency of these men as something very commendable in them and as peculiarly their own. In boasting of it they call it "*liberality*," whereas it is only stupidity or ignorance. Stupidity, in not seeing that the difference between them and those who obey law is not one of less interest in the cure of the sick, but of a better knowledge of better means for their cure; and ignorance on the part of these *liberal* gentlemen of the way of law and its resources. When they plume themselves on this so-called liberality, which will not "let patients die," etc., they seem to think and to intimate that there are those who are less *liberal*, and who would thus let them die. Who are they? We have never met such an one. We will only add of these who thus claim liberality beyond others, that they have, in our observation, not been found to be the most successful healers. Those have been the most successful who in the discharge of their first duty—finding the curative—have most perfectly complied with the requirements of our law.

---

**Medical Times, loc. sit.*

And their success has been greatest when, having thus performed their first duty, they were mindful of its logical corollaries in their administration of the curative. It is not enough for the best success in practice that the specific be *found* ; in addition to this, it must be *rightly used.*

There is no infallible law to lead us in this, as there is in the finding. But there are certain rules and considerations necessary to be observed by him who will be content with nothing less than his whole duty. The nature of the case to be cured will be an important element in determining the management of the administration of the specific. If it be rapid in its destructive progress, the method with the remedy will be different from that required by a case where this is slow. If violent in its attack, it will be different from that where the attack is insidious or more moderate. If the patient be very susceptible to the action of drugs, it will not be the same as with one where this susceptibility is more dull and obtuse. And yet there are rules which are applicable to all cases, whatever their nature.

The first is, the specific being *one*, this and this alone is to be given, and its action in no case to be interfered with by the presence of other drugs, or by any proceeding which can by possibility interfere with its specific action, *i. e.*, the corollary of the law, which declares *one remedy at a time*, is to be obeyed implicitly. This corollary disposes of all so-called adjuvants and alternations, and stamps them as contraband in practice in all cases and at all times. Their presence in any treatment of the sick deprives the true specific of its homœopathic character. This is in the very nature of the case, and is perfectly apparent to only a very moderate acquaintance with the true philosophy of disease and drug action, *i. e.*, with the philosophy of Homœopathy.

The second is, while the dose already given continues to act, give no more. This is only other words for while improvement continues to progress, after any given dose, there is to be no repetition of it, nor is any other remedy to be given while this progress continues. These rules are as old as Homœopathy, and are indispensable to its best practice. If there be doubt whether improvement is progressing the dose is not to be repeated. Wait, and the doubt will resolve itself. To be in haste to repeat and so violate this rule is to haste to do mischief.

Then the nature of the case to be cured is an important factor in guiding our judgment in the management of the specific. Violence of attack and rapid progress of the destructive process are to be met by a different method from that which serves best in cases of an opposite character. If the case be pneumonia, and the initiation be with chill, followed by great heat and restlessness, with rapid and moderately hard pulse, oppressed breathing, and dry, hard cough, the treatment may begin by dissolving a half dozen pellets of the proper remedy in two-thirds of a tumbler of pure water, and of this a teaspoonful may be given every ten minutes for the first hour, every twenty minutes the second hour, and every thirty the third, and then every hour, remembering always to stop the doses whenever free perspiration appears, with relief of the pains, cough and difficulty of breathing. This method, in these circumstances, will often cut the attack short and virtually effect a complete cure in an incredibly short time. It is appropriate only where the febrile reaction of the case is sharp from the beginning, and is only to be practiced in the earliest stage of the attack. In the later it will be wholly unsuitable and probably mischievous. This permits the remark that the management of the remedy may be altogether different in the different periods of the progess of the same disease. It will probably be found of the rarest that pneumonia will be cut short after the initiatory stage has passed, though this may often be effected in this stage by the method given above.

In diseases from blood poisoning, which rapidly exhaust the life power, a similar administration of the remedy may be followed by rapid curative results, for example, in malignant cholera in the outset, and in its progress it may be needful and beneficial to repeat the doses at very short intervals till the disease is arrested, or, in the later stage, reaction is established. Then, as in pneumonia, cases of this disease come under the general rule—no repetitions while improvement progresses.

Acute and chronic diseases, though alike under certain general rules as to repetition and management of doses of the curative, require, often, different methods as to particulars if the best results are to be secured. For example, in acute disease of a different character from pneumonia as to violence of attack and rapid progress of the process of destruction, take a case of typhoid fever: The attack is insidious in the beginning. There may be only listlessness, disinclination to all activity, drowsiness, moderate pains in the head, with vertigo, debility, loss of appetite, with slight chilliness on motion, etc. The whole group is not evincive of violence of assault, though sufficiently indicative of grave sickness. To treat this group with the rapid repetition of doses so often triumphant in pneumonia will not only fail of any benefit, but may go far to insure a fatal termination of the case.*

The group is significant of torpor and not of excess of action. The case may require repetitions, perhaps many of them before reaction from this is secured, but not at the short intervals which are often best in the very early stage of acute inflammation, as in pneumonia or croup. The torpor of vitality in the typhoid state may delay visible response to the imprèss of the specific till this has been given many times, but this delay is, in itself, no warrant for a change of remedy or for interference with its proper action by any so-called "*inter-current*" remedies, or by any disturbing resorts whatever. The best results will come from a strict adherence to the selected remedy, always taking it for granted that this *is* the *right*, *i. e.*, the homœopathic remedy to the case. If it be so, and we assume a sufficient intelligence on the part of the practitioner to decide this question, then by patient and persevering adherence to it the best possible practical results are sure to follow. If the doctor does not know enough to assure himself that he has selected the right remedy, he has undertaken a duty when he assumed the treatment of this fever for which he is wholly unqualified. And to give to such an one rules for the management of the right remedy,

---

*We have to-day listened to an account from a doctor of how he was treated by another doctor for what was called "Malarial Fever." The doctor divided the twenty-four hours into three periods of eight hours each. The first of these he called the *chill* period, and through these eight hours he gave his patient a dose of Nux Vomica every thirty minutes. The next he called the *heat*, and through it he gave Arsenicum every thirty minutes. The third was the *sweat*, and through this the doctor got China every thirty minutes. So he got forty-eight doses of medicine in twenty-four hours, no one of them, as far could be learned, having any legal relationship whatever to the case. And yet the community in which this doctor lives and practices regard him as a homœopathic physician, because, forsooth, he tells them he is one. There can be no other reason for their so regarding him, if this be a fair specimen of his dealing with the sick. No old school doctor could have done worse, nor in clinical use of drugs could he have gone further from all which is characteristic of a homœopathic practice. This was a year and a half ago, and the patient has never been well since. Are there words which can properly express the disgust such stupidity, ignorance and foolishness excite?

when he does not know whether he has found it, can be only to waste time and words. But even he may understand that doses which are too large or too often repeated, and for too long a time, may crush out of the poor patient his last and only chance of life. This takes for granted that the doctor blundered on to the true specific for his case, which, acting *like* and in the direction of the action of the morbid cause, adds so much to the force of this as to exhaust the remainder of the life-force which had survived the impact of this cause. This is true of the treatment of all grave acute diseases, the initiation of which is characterized by a stealthy approach.

Where diseased action is ushered in by *violence* of symptoms the case is different by reason of the rapid absorption or exhaustion of drug power, so that there is no accumulation of this to the detriment of the patient, though the dose be repeated at short intervals and many times. The best management of the dose in acute diseases must come largely from the good judgment of the prescriber and his ready discrimination of the degree of violence and progress of his case, he always remembering that *rapid* repetitions are only in place in the very *earliest* stage of acute inflammations and in collapsed conditions, as in malignant cholera, and that this should cease on the first appearance of the yielding of this violence in the one case or of reaction in the other, and these doses are only to be repeated when there is evidence that the action of those already taken is exhausted, and then, after this, repetitions are to be strictly and only *pro re nata, i. e.*, not because so many minutes or hours have elapsed since the last dose, but for evidence that this has ceased to act. There is no dogmatic rule for this duty. It is always and only a duty to be controlled by judgment, care and discretion. There is one rule which, in these cases of acute disease, is always safe for a wise man to follow. It is—if there be a *doubt* as to repetition, don't repeat. A little time will solve the doubt, and this loss of time is as nothing in comparison with the mischief of inopportune repetitions. In this we are fully assured more and more mischievous mistakes are made than by any other fault of practice. So much depends, in treating acute cases, on the matter of repetitions of doses and the sound judgment of the prescriber, as to make the duty of cultivating his power of perception of change and progress for better or worse, and of violence of morbid action, one of the very highest importance. The true decision of the question of *right* repetition depends so largely on this, and successful treatment so largely on *right* repetition, that no endeavor to cultivate this power can be greater than the importance of its possession. The rule is, repetitions are to be in the direct ratio of the violence of acute attack of inflammatory diseases, or of the degree of torpor in diseases characterized by collapse, and to be continued till there is evidence that the life-force has responded to the presence of the specific in the improved condition of the patient, *i. e.*, the greater the violence or torpor of a given case the more frequent will be the repetitions.

Then, further, these factors of violence and torpor, however great, are not to be regarded as calls for massive doses of the specific—quite the contrary. If this mistake be made, it may happen that the very agent ordained of God for the cure, and rightly selected by the intelligent prescriber, becomes by reason of its excessive presence the *ally* of the morbid cause because of its similar action, instead of acting as its curative, as it would had it been present only in its appropriate quantity. The erroneous idea that violent action of diseases require massive doses of their curative for their healing has destroyed many lives—the Almighty only knows how many; but even if loss of life do not follow this error, it can never fail to embarrass—and greatly—the subsequent treatment of the case to and through its convalescence. It should never be forgotten that it is the specific impress of the drug which cures, and not—nor more certainly ever—because of the presence of massive doses of it. If the drug be really like, it is true, and all sound observation and experience will confirm the statement of Hahnemann, that the dose cannot well be too small. There is no truth in the rule given the writer in his early homœopathic experience—"Acute diseases require low numbers for their cure, and chronic high." The 30th centesimal was then *high*, and the highest. It is now beyond all doubt that many of the most acute and violent diseases are cured more speedily and more perfectly by high than by low numbers. This was clearly witnessed in an epidemic of dysentery which prevailed in Brooklyn more than thirty years ago. I studied it with all possible care, and every study brought out the same remedy and would give me no other. Haynel was then my neighbor and friend, and he studied it with me, and said I had found the remedy. But my cases dragged along, instead of finding prompt relief from this drug. I had just at this time of Haynel's help a patient who had baffled me and my drug for a fortnight. She got doses of 30th, 15, 12, 3, and 1, and was not relieved by either. I had a few pellets of the 400th of this drug of Jenichen's preparation, which I kept as a curiosity, and not for use. I determined to give this to this patient who had caused me so much trouble and who had gone through so great sufferings. I put two of these pellets in two-thirds of a tumblerful of water and gave her a teaspoonful at nine o'clock A. M. I saw her again at one o'clock, and she received me smiling, saying, "Why, doctor, that last dose you gave me acted like a charm." And so it had. She required no more medicine. All my subsequent cases in this epidemic were cured thus promptly by this 400 of Jenichen. If experience proves anything, mine in this epidemic proved that the choice of the potence for the cure is not a question of indifference. Some have said, "I don't care as to this matter of potence if I can only have the simillimum." No doubt this is the first and most important question the prescriber has to decide, but it is not the only important one by far. And the rule for the choice of the potence is —*The more like the remedy is to the disease the higher is to be the dose.* Then the *management* of the remedy in acute diseases is made up of choice of potence, repetition of doses, and the absence of all other drugs than the specific and of all other disturbing causes, and as a prime duty, the prescriber is never to let impatience, his own or of others, run away with his judgment.

Then it is not a matter of indifference the form in which the dose shall be given. As a general thing, if pure water can be had, it is better to give the medicine dissolved rather than dry. I believe the solution acts more speedily, profoundly and persistently, and is less liable to disturbance from contingencies.

Another rule as to diseases characterized by periodical paroxysms —give the medicines between the paroxysms. This refers to intermittent fevers, epilepsy, etc.

Still another rule in treating both acute and chronic diseases is, having found your simillimum and given it, it is not to be changed for any other remedy for any reason other than a change of symptoms, by reason of which this has ceased to be the simillimum for the case. There is no greater error in practice than frequent change of remedies, though the motive may be a desire for a greater good to the patient. It is only mischievous and sometimes fatal.

The general rules for managing the dose of the simillimum is the same in chronic as in acute cases. In the details, or in certain particulars, there may be a difference. In chronic cases it is often best to give a single dose and let it alone, being in no hurry either to repeat it or to give another drug because the improvement is not realized as soon as was expected. The best results sometimes follow long-delayed amendment. This was illustrated in the case of my first patient in Brooklyn. She was about thirty years of age. Both her parents had died of phthisis when she was a young child. She moved into a new house before the plastering was dry and took a severe cold, with fever, cough, pains in the chest, oppressed breathing, and great obstinacy of disposition. After the fever was in a measure subdued the chest symptoms remained, not relieved at all. They were *worse in the morning hours*, and the expectoration, pains and oppression answered to *Calc. carb*. This morning aggravation decided the choice of this remedy. Jenichen's high potencies had then been brought to our knowledge by the elder Gross in a paper in *Das Neues Archiv*, 1844, which I had just translated. In this he gave a series of cases treated by these potencies, with results altogether new and surprising. I had a few pellets of the 2000th of this remedy, and, inspired by these reports of Gross, decided to give my patient *two* of them dry on her tongue. In a short time she was seized with a violent fit of coughing, far more severe than any she had had before, and this was repeated at short intervals during the afternoon, night and the next morning, the morning paroxysms being most violent. At my morning visit I found she had had a wretched night, and she was now greatly exhausted by coughing and sleeplessness. I told her this new violence was the effect of the dose I put on her tongue the day before. She replied she knew it was. She was told if she would be patient and bear the assault, reaction would come and she would be better. She said it was not necessary to talk about patience, in the matter of interfering with this aggravation by attempting its relief by other remedies, for she had determined to take no more medicine of any kind whatever; so let what might come, she would let it take its own course. After the seventh or eighth day, she remarked—and each day after, when I made my morning call—"Doctor, I think the reaction is a long time coming."

And so it was, for till the morning of the sixteenth day she bore her suffering like a martyr. But then it did come. The whole train of her troubles passed away like a shadow, and her recovery was prompt and perfect. This is the more remarkable, as it was the third time I had been successful in rescuing her from attacks which threatened to end in her family malady.

There are those in our school who deny drug aggravations of symptoms of sickness. They say they have never seen them, and therefore do not believe they are ever seen by others. This is not good logic, and can hardly be accepted as a reply to such an experience as this of my very intelligent and obstinate patient. It is more than likely her obstinacy saved her life this time. If the dose had been interfered with by attempts to subdue its violent action by other medicines, the error would in all probability have been fatal. Her doctor might not have had the knowledge, faith and firmness to wait sixteen days for a reaction which gave, in all this time, no sign of its approach. It was a long time to wait and watch and witness sufferings tempting so greatly to efforts for their relief by other remedies. There was no merit in his waiting. The patient made it compulsory and the compulsion made it a success. I have ever since regarded this as one of the most instructive cases in my clinical experience. It served as a lesson, the moral of which is an important one, because it contains a principle. It is this: being *sure* of your remedy, time is no authority for repetition or change because curative reaction is delayed even a long time after its dose has been given. The simillimum will justify itself *if it be let alone.* Being thus *sure*, there need be no nervous anxiety because of delay in signs of visible improvement.

This case well illustrates, also, the dealing with the dose where this is given dry on the tongue, and all at one time. There is another method which dissolves the dose in water and gives it in divided portions at definite intervals of time. This method is well-represented in a prescription made by that master of prescribers, Bœnninghausen, for the writer of this paper in 1858. At his request the symptoms of a succession of very painful attacks, from which the writer had suffered at indefinite intervals of time, were written out and given to this great master of our art. He, in the plenitude of his great practical power and experience, gave a whole day to the study of this record. Prescribing, with him, was never a duty performed in a hurry. He studied every case before he selected its curative. At the age of seventy-six years this was as true of him as it had ever been. At the appointed hour, when I called on him, he named the required remedy with the greatest confidence, and directed that *two* pellets of the 200th centesimal potency of the medicine be dissolved in *eight ounces* of distilled water, to which, for its preservation, a teaspoonful of French brandy should be added, and a *teaspoonful* of the water to be taken each night on retiring. And he added, "you need have no fears of the result. It will cure you, sure. After this has run its course you may require a dose of [naming a second medicine] to complete the cure. After the first dose you may have a paroxysm, but if you do you will never have another. After you have taken this two or three times you will feel very different." I did not prepare the dose till I came to Berlin, where I was to remain some days. I took the first dose on retiring, as directed, at 10 P. M. Just as the clock was striking twelve that night I was awakened from a sound sleep by my old pain, and I think it was the most severe I had ever endured. I continued the dose on retiring each night for about two weeks, when I felt so much better I threw the bottle away. During this time I was subjected to the fatigue and vicissitudes of travel, and it was not until I had been in Rome about a week that I felt I was getting sick, and thought myself threatened with Roman fever, and determined to leave the city the first conveyance which would carry me away. In this state I remembered the old master's second-named medicine, and took a single pellet on my tongue. The next morning I was as bright as ever and remained in the city till I had accomplished my object in visiting it. I have never had another paroxysm of those pains since that which followed my first dose, now twenty-four years. I have always regarded this prescription as the most remarkable example of professional sagacity I have chanced to meet or to have read of. It also exhibits well a second method of managing the dose in chronic diseases, which often will master great difficulties in a very surprising manner. This method may be found preferable to the dry in cases of any constitutional cachexia, where it may be desirable to pervade the life power and functions of the patient with the medicinal power in the most thorough manner possible. It was often practiced by the elder Gross with the best results.*

---

* *Neues Archiv*, b. 1, th. 3, s. 35., *et seq.*, and b. 2, th. 1, s. 38, *et seq.* The series of reported cases of cure here referred to were those which brought us our first practical knowledge of the greater curing power of what we have come to designate as "*the high potencies.*" Perhaps there has not been a more remarkable series of cures reported in our literature. No man has been more astonished than was Gross, when he discovered the greater curing power of these potencies as compared with the 30th (which, up to this time, had

His method was to dissolve a single pellet in about four ounces of water, and give the patient a teaspoonful once in twenty-four hours. From the experience of such masters as Bœnninghausen and Gross we learn this most important fact: that great sicknesses and danger to life do not require for their cure great quantities of the simillimum which cures. To mistake in this, which is so natural and easy, and give massive doses, is little less than assured failure, and too often will only result in increase of suffering and danger to the patient. It is the first and greatest practical error—to *give too much*—and the second is like and of near kindred of it—to *give too often.* Either of these may spoil the success of the best selection of remedies, and either of them will certainly be found injurious by whoever is so ignorant or unwise as to allow these most fatal of blunders in his practice. In this series of cures by Gross, to which we have referred, are found cases treated with dry doses. Quantity, *one pellet* on the tongue. Time of repetition or of substitution—he often waited two weeks for result from his dose if this was not manifested earlier, and this waiting was justified by complete success. Grave sicknesses and dangers call for *right* remedies, and not for *massive* doses of them.

---

been the highest in use by him or by any one) or lower numbers. In this series of cures he used the 200, 300 and 400 oftener than higher numbers, and rarely any higher than 2,000. The so-called "*highest potencies*" had not then been developed. There cannot but be interest in conjecturing a practice with these by so accurate and acute a prescriber as Gross.

---

